# Severe Cervicofacial Cellulitis in Pregnancy- A Review of 18 Cases

**DOI:** 10.22038/ijorl.2019.34909.2154

**Published:** 2020-03

**Authors:** Kevin-Uchenna Omeje, Ifesinachi-Joy Omeje, Rowland Agbara

**Affiliations:** 1 *Department of Oral and Maxillofacial Surgery, Bayero University, Kano and Consultant, Oral and Maxillofacial Surgeon, Aminu Kano Teaching Hospital, Kano, Nigeria.*; 2 *Department of Obstetrics and Gynaecology, Aminu Kano Teaching Hospital, Kano.*; 3 *Department of Oral and Maxillofacial Surgery, College of Health Sciences, University of Jos, Plateau state, Nigeria.*

**Keywords:** Cervicofacial, Infection, Pregnancy, Tooth

## Abstract

**Introduction:**

Cervicofacial cellulitis can be rapidly complicated with a difficult airway when prompt management is not instituted. It may have some serious consequences for developing baby when a pregnant woman is involved. This study presented the experiences gained from the management of cervicofacial cellulitis in pregnant women.

**Materials and Methods:**

The present study was conducted on 18 pregnant women with cervicofacial cellulitis affecting more than one facial space at the presentation in a regional tertiary hospital within a five-year period (January 2013 to December 2017). The collected information included patient age, clinical diagnosis, number of involved facial spaces, gestational periods, and duration of hospital stay. The collected data were analyzed using SPSS software (version 15.0).

**Results:**

A total of 131 patients with cervicofacial cellulitis were admitted during the study period, out of which 18 patients met the inclusion criteria and were investigated in the present study. These 18 patients were within the age range of 20-43 years with the mean age of 29±7.1 years. There was a statistically significant relationship between the number of fascial spaces involved and duration of hospital stay (P=0.04). All the patients had incision and drainage of the affected facial spaces under local anesthesia with good outcomes.

**Conclusion:**

The prompt management of pregnant women with cervicofacial cellulitis in a multidisciplinary manner is important to ensure uneventful outcomes in the lives of both the pregnant woman and unborn child.

## Introduction

Cervicofacial cellulitis usually refers to a rapidly progressing infection of the fascial spaces of the head and neck which is usually of odontogenic origin and can be quickly complicated with a difficult airway when prompt management is not instituted (1,2). The fascial spaces that are often primarily implicated include the submandibular and sublingual spaces (1). The involvement of the submental space often follows lymphatic spread; however, the involvement of the parapharyngeal spaces are from secondary communication. The bilateral and simultaneous involvement of these primary spaces is referred to as Ludwig’s angina (1).

Ludwig’s angina is a rapidly progressive infection, often fatal with a potential for poor outcomes. There has been a lot of debate regarding the actual path mechanism of these infections; nevertheless, it is generally accepted that the native oral streptococcal bacteria or mixed aerobic and anaerobic organisms in the oral flora elaborate enzymes, such as hyaluronidase and chondroitin phosphatase break down connective tissues and permit the rapid spread of this infection (4).Pregnancy is a physiologic condition with a significant alteration of the immune functions. Pregnant patients are not treated as immunocompromised hosts; nonetheless, it is known that maternal immunity is suppressed in response to the fetus (4).The immune status of pregnant patients may be further affected by nausea and vomiting, which are common in pregnancy and may lead to nutritional anemia, especially in patients with hyperemesis gravidarum. Pregnant patients also have a decrease in cell-mediated immunity and natural killer cell activity. Gupta et al (5). noted that in pregnancy, when medical and surgical treatments are considered, both the physiologic changes of pregnancy and perinatal effects of the treatment should be put to perspective. The need for the consideration of the fetus in the surgical and chemotherapeutic decisions makes the management of these patients challenging. The high potential for morbidity and mortality in cervicofacial cellulitis is often related to the persistent threat to airway (5). This becomes an important concern when fetal hypoxia is considered during the periods of hypoventilation. There is a need for a robust multidisciplinary approach for the management of pregnant women with cervicofacial cellulitis to ensure uneventful outcomes. The positional disadvantage of these pregnant women relative to nonpregnant patients when reclined on the dental chair, especially as they approach the third trimester makes the institution of care challenging with inexperienced hands.

Our institution serves as a referral center to many secondary and tertiary healthcare centers within the region. The need for the adoption of a working protocol based on the experiences gained over the years from the management of these cases helps to maintain a reproducible positive treatment outcome and improve the quality and consistency of teaching methods as well as training. Gupta et al (5). In a literature review noted that there was a paucity of data on cervicofacial cellulitis in pregnancy. The present study highlighted important findings obtained from 18 pregnant women treated for severe cervicofacial cellulitis.

## Materials and Methods

This retrospective review was carried out on patients who presented with facial cellulitis to the Oral and Maxillofacial Surgery Unit of a regional tertiary hospital within a five-year period (January 2013 to December 2017). The subsets of those who were pregnant and had cervicofacial cellulitis affecting more than one facial space at admission formed the study cohort. The sources of retrieved information were patient case files, outpatient clinics, accident and emergency registers, as well as inpatient admission records. The study protocol was approved for ethical clearance by our Institutional Ethics Committee. 

The retrieved information included patient age, clinical diagnosis, number of involved fascial spaces, duration of hospital stay, gestational periods (computed as trimesters), attendance at antenatal clinics, and source of infection. Other retrieved data were previous medical and obstetrics/gynecological history, review by the obstetrics and gynecology unit, surgical and chemotherapeutic treatments, as well as employed imaging modalities. The collected data were analyzed using SPSS software (version 15.0). Categorical variables were presented as frequencies and percentages; however, continuous variables were reported as mean and standard deviation. Comparative statistics were determined using appropriate statistical tools, and p-value less than 0.05 was considered statistically significant.

*Institutional protocol for the management of severe cervicofacial cellulitis in pregnancy*

These patients presented to our unit mostly through the accident and emergency or oral and maxillofacial outpatient clinics. They were considered emergency cases as they often presented acutely. Following history taking and examination, they were admitted into the ward, and the administration of parenteral antibiotics commenced. 

Obstetrics and gynecologic reviewsareusually sought before incision, and drainage/ decompression is performed under local anesthesia (LA). However, in cases where the patient life is at risk due to airway obstruction, incision and drainage are carried out before obstetrics and gynecologic reviews. The reasons for prompt gynecologic/obstetric review are to determine the status of the pregnancy and fetal well-being.

The site of incision and drainage is determined by the affected facial space. Following extra-oral incision, sinus forceps are inserted into the fascial space involved in line using Hilton's method. Then, a corrugated rubber drain is placed and secured for continuous drainage. The positioning of these patients is an important consideration as the usual retroclined position used for nonpregnant patients on the dental chair may cause the gravid uterus to compress the inferior vena cava, diminishing venous return. 

This results in supine hypotension syndrome with bradycardia, hypotension, and syncope, especially on standing up from a seated position. The resultant effect is tissue hypoxia with consequent diminished placental perfusion, which may have a deleterious effect on the fetus. Therefore, it is important that the patient is seated with her right hip elevated 10-12 cm or lies in the supine position with a 5-15% tilt on her left side to reduce the pressure on the vena cava (7-10).Where hypotension is not relieved by these measures, the patient should be asked to acquire a full left lateral position (10). The source of the infection is often determined at the time of clerking and examination and if odontogenic in origin, the extraction of the offending tooth is carried out as soon as possible. 

## Results

A total of 131 patients with cervicofacial cellulitis were admitted intoour facilityduring the study period, out of which 21 patients were pregnant at the time of presentation. However, only 18 pregnant patients met the inclusion criteria (the remaining three pregnant subjects were excluded since only one fascial space was involved in these patients). The distribution of pregnant patients by age and involved facial spaces are represented in Table 1. The patients were within the age range of 20-43 years with the mean age of 29±7.1 years; however, the duration of their hospital stay ranged from 5-21 days with a mean value of 10±3.4 days. 

**Table 1:** Age distribution of patients by facial space involvement

**Facial Space** **Age (years)**	**2 spaces**	**3 spaces**	**4 spaces**	**>4 spaces**	**Total**
20-25	1		2		3
26-30			3	1	4
31-35	1	2	1	2	6
36-40		1	1	1	3
41-45		1		1	2
Total	2	4	7	5	18

All the patients withcervicofacial cellulitis involving more than four spaces were admitted for more than 10 days (Fig.1a). There was a statistically significant relationship between the duration of hospital admission and number of involved fascial spaces (P=0.04). The majority of the patients (n=12; 67%) were in their third trimester of pregnancy; however, four and two patients were in the second and first trimesters, respectively.

**Fig 1(a):**
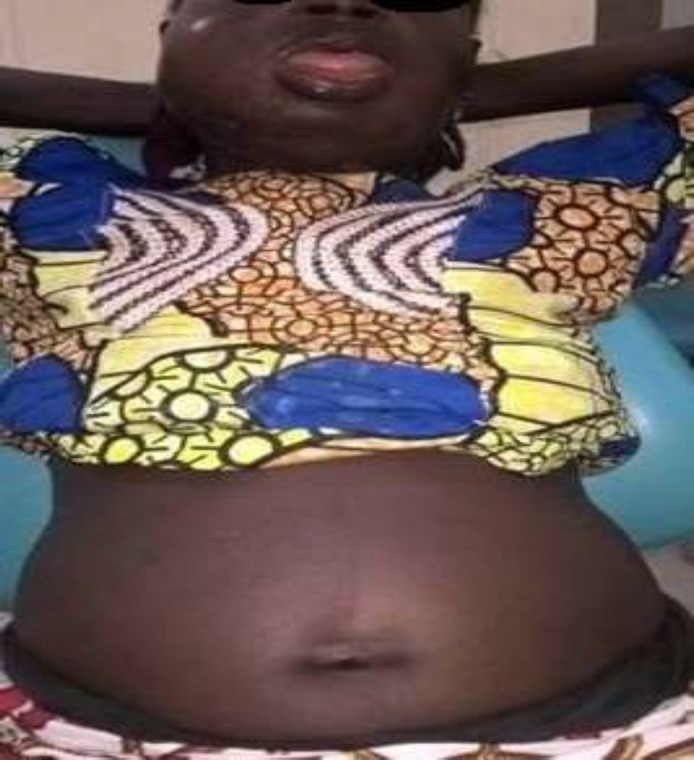
Involvement of multiple fascial spaces

The source of infection for all the patients was odontogenic. Only two subjects had a secondary comorbid medical condition (one of them was diabetic, and the other was both diabetic and hypertensive); nevertheless, both had good control. Most of the subjects (n=16; 89%) attended antenatal clinics at the time of diagnosis. The status of the remaining two patients was not documented regarding the attendance at the antenatal clinic. 

All the patients were firstly reviewed by the obstetrics and gynecology team within 48 h of admission. Furthermore, they received 1.2 g intravenous augmentinevery 12 h, 500mg metronidazole every 8 h, and 300mg intramuscular paracetamol every 8 h. However, the subjects who were in their first trimester of pregnancy were given intravenous ceftriaxone without metronidazole. Each patient had incision and drainage of the affected fascial spaces under LA, and corrugated rubber drains were placed except for five patients where there was no record regarding the type of utilized drain. Because pus specimens were not collected for microscopy, culture, and sensitivity at the time of drainage, no culture result was available for these patients. None of the treated subjects required the secondary exploration of the involved fascial spaces after the initial drainage. The extraction of the implicated teeth was conducted for all the patients before discharge (eight patients had extraction at the time of incision and drainage), and none of the subjects required a surgical airway for their management (Fig.1b).

**Fig 1(b):**
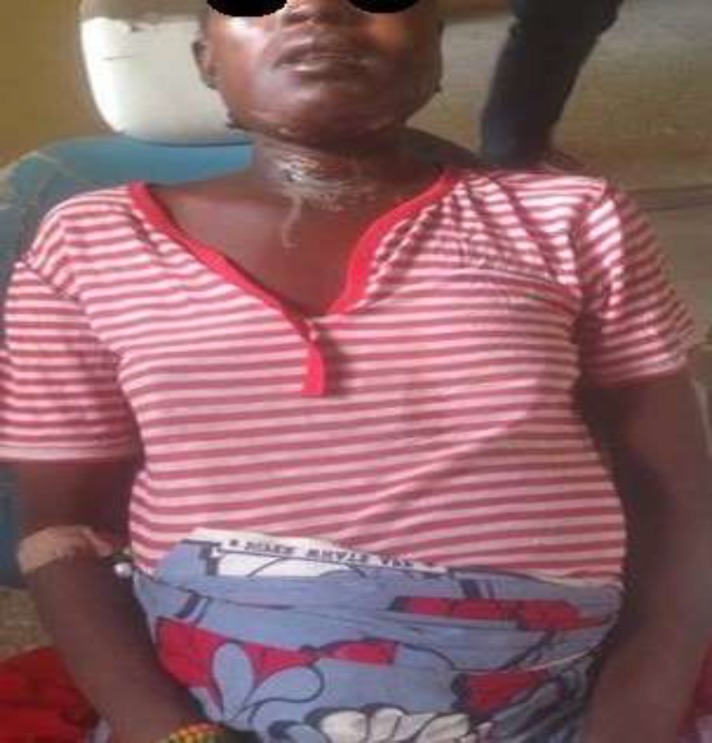
Significant Resolution of swelling following drainage without the need for surgical airway

## Discussion

The anatomy of the oral cavity plays a significant role in the spread of cervicofacial infection. It has been reported that 75% of these infections are usually of odontogenic origin(4).The infection of mandibular teeth whose root apices are below the mylohyoid muscle tends to cause cellulitis, which begins at the submandibular space; however,the infection of mandibular teeth whose root apices are above this muscle causes cellulitis, which starts from the sublingual space (5). The latter leads to the edema of the mouth floor and rapid airway compromise.There is usually an upsurge in the levels of estrogen and progesterone in pregnancy. These hormones are largely responsible for marked physiological changes observed in pregnant women. The combined effect of these hormones is responsible for the increased susceptibility to irritation from dental plaque in pregnancy leading to gingival tissue inflammation and bleeding at the slightest perturbation (11,12). Pregnant women are also at a much higher risk of infection, compared to nonpregnant women (4,5). This susceptibility to infection in pregnant women may be related to diminished immune response resulting from decreased neutrophil chemotaxis, cell-mediated immunity, and natural killer cell activity among them (13,14).

A prevalence rate of 13.7% noted in the present study is higher than those reported in previous studies (15,16) This may be related to the fact that most of the patients in the present study were mainly from poor rural areas in Nigeria where malnutrition may be a major issue, potentiating the progression of dental infection in the background of pregnancy. The duration of hospital admission in the present study was observed to increase with the increasing number of involved fascial spaces at the presentation, and this finding is in agreement with previous results of the general population as reported by Fomete et al (17). This may be related to fact that most of the patients with the involvement of many spaces were more likely to have the involvement of the deep neck fascial spaces in addition to superficial spaces. Increasing fascial space involvement is likely to be associated with airway compromise, constitutional symptoms, and malnutrition due to odynophagia. All these factors may contribute to delayed recovery.

Adequate and prompt care for these patients is essential as two individuals (i.e., pregnant patient and unborn child) are at risk of serious complications. The pathomechanism of these adverse outcomes may not be unconnected with the activities of gram-negative anaerobes, which are known to produce endotoxins and lipopolysaccharides. They elaborate local inflammatory mediators and consequently play an important role in the progression ofcervicofacial infections. The resultant pro-inflammatory cytokines have been noted to be responsible for the placental modifications that bring about pregnancy-related complications (5).The age of the youngest patient in the present study was 20 years that may reflect the young age of childbirth in the region of study, compared to that reported by Osunde et al (18). Pregnancy at a very young age is often complicated with anemia more than the older age groups. Anemia in these patients may limit the ability of the subjects to withstand infections.

Although the majority of the patients in the present study were in their third trimester of pregnancy and underwent treatment within the same period, Wong et al. (15) are of the opinion that minor routine dental treatments should be avoided during the first and third trimesters. Nevertheless, they should be carried out during the second trimester of pregnancy. However, they noted that emergency treatment should be instituted irrespective of the period, and a delay or avoidance by either the patient or clinician is often responsible for severe spread.

Tooth extraction is an integral component of treatment for the cervicofacial cellulitis of odontogenic origin; however, it may act as a portal of entry for microorganisms. The resultant inflammatory mediation may set the stage for systemic inflammatory response culminating in fetal distress (19). The implicated microorganismal agent may enter the bloodstream and subsequently lead to intrauterine infection and associated adverse pregnancy outcomes, which may result in preterm birth, low birth weight, fetal growth restriction, pre-eclampsia, and miscarriage (20).This possibility of spreading infection justifies the need for antibiotic cover prior to tooth extraction, especially in pregnant patients who may have altered immune functions. The socket of the extracted tooth often forms a part of drainage access for the infection resulting in the improvement of the decompression process. The factors that determine the time of this extraction include the obtainable amount of mouth opening, stability of the patient to undergo an extraction, and experience of the surgeon. 

The mouth opening is often limited in cervicofacial cellulitis and results from muscle spasm that is considered a protective mechanism to limit the spread of infection. The mouth opening often improves when the administration of antibioticsis commenced and drainage instituted. However, at presentation, some patients are usually in distress and uncooperative due to respiratory distress. The extraction for such patients may defer until when they are stabilized. 

There are various techniques for tooth extraction in subjects with cervicofacial cellulitis. The surgeons who are conversant with these techniques may be able to extract the offending tooth at the time of initial presentation, especially in cases where tooth extraction is expected to play a role in the decompression of the cellulitis. The variation in the time of extraction noted in this study was related to adequacy of mouth opening at the initial presentation.

The drug of choice during pregnancy is an important consideration in the management of these patients. This informed the United States Food and Drug Administration to classify drugsduring pregnancy into five groups (i.e., A, B, C, D, and X) based on their toxicity. Penicillin was the preferred drug of choice, and this finding is similar to those of previous studies both in the general population and pregnant patients (17,18,21). Penicillin is a class-A drug and is widely available/relatively cheap in this environment. However, its wide availability and affordability are associated with frequent abuse. Tetracycline and aminoglycosides are often contraindicated in pregnancy due todiscoloration effect on teeth, effect on the developing bone, and ototoxicity, respectively) 10.( Although the use of metronidazole is relatively contraindicated due to its potential teratogenic effect, it is administered when the benefit is deemed to outweigh the risk )^10^).

Analgesics should be cautiously used in pregnancy. Common analgesics, such as aspirin, should be avoided, especially in the third trimester due to their association with delivery complications, particularly postpartum hemorrhage. Nevertheless, nonsteroidal anti-inflammatory drugs are generally contraindicated in the same period due to their ability to cause a delay in the onset of labor and premature closure of the ductus arteriosus leading to right-sided heart failure and consequent fetal hydrops (10). Opioids may be dangerous, especially within the last few hours before delivery because the metabolite may still be in the fetal circulation at the time of delivery resulting in respiratory depression.

In the present study, pus specimens from the patients were not sent for microscopy, culture, and sensitivity. This is because these subjects in most cases had commenced antibiotic therapy for several days before the presentation. Moreover, anaerobic culture was not possible in our facility during the study period. The abuse of antibiotics by patients with cervicofacial cellulitis prior to presentation in this environment has been previously highlighted by Fomete et al (17). Poorly controlled comorbid medical conditions have also been associated with poor prognosis in pregnant women with cervicofacial cellulitis. Osunde et al. (18) noted overwhelming sepsis in a pregnant patient with uncontrolled diabetes mellitus as the cause of mortality in one of the studied patients. The only two subjects in the present study with comorbid medical conditions did not show any difference in the outcomes, compared to other patients probably because their medical conditions were controlled.

The relevance of pregnant women attending antenatal clinics and probable relationship with the prognosis of cervicofacial cellulitis are worthy of note. The majority of the patients in the present series attended antenatal clinics, and they had an improvement in their clinical status resulting in the eventual discharge. This finding is different from that reported by Osunde et al. (18) who recorded 20% mortality in the studied cases. The result of nonattendance to the antenatal clinics in the majority (80%) of their patients may have contributed to this poor outcome. Pregnant women are monitored during antenatal visits to quickly stop and abort any untoward events. Some of the parameters monitored during these visits include hematocrits, adequate nutrition, and other medical conditions. The consideration of all the aforementioned factors ensures safe pregnancy and delivery.

Additional findings in the literature that have been noted to improve prognosis in these patients are the delivery of the neonate, especially in cases where there is an obstetrics indication (22). Delivery is indicated in cases with nonreassuring fetal tracing, which may be due to fetal hypoxia or acidosis (22). Maternal health status has been reported to be enhanced immediately with such delivery. The physiological changes associated with this delivery are said to improve ventilation status. The increase in functional residual capacity and respiratory compliance following a downward displacement of the diaphragm, which occurs postpartum, has been noted to enhance gas exchange leading to the reduction of hypoxemic state. 

Although Houghtet al. have strongly advocated elective tracheostomy under LA for these cases following their exhaustive review of literature citing about 75 cases, none of the patients in the present study had a tracheostomy (23). A tracheostomy is only necessary where routine incision and decompression are ineffective in relieving the upper airway obstruction. A tracheostomy in inexperienced hands may be challenging, especially due to the already distorted neck anatomy following cellulitic changes. There is also a risk of the dissemination of the septic focus by opening up and contaminating fascial planes through the neck resulting in possible mediastinal invasion (24). Imaging investigative modalities in pregnancy, though not absolute contraindications, should be used with caution, especially in the first trimester during which organogenesis occurs (25). The potential for fetal abnormalities to be caused by irradiation in utero is greatest in the third to eighth weeks of pregnancy when cells are largely pluripotent rather than being totipotent. 

Threshold doses for deterministic effects on the developing fetus are within the range of 100-500mGy (25). It has been demonstrated that the irradiation doses of less than 5-10 cGy, which are less than average irradiation dose required for these cases, are not associated with the increased risk of development of congenital defects. A single orthopantomogram (OPG) usually will provide sufficient information at an acceptable irradiation exposure (26).

Althoughcomputed tomography (CT) scan produces an acceptable irradiation dose of less than 10cGy, it provides an exposure higher than the noted dose with an OPG; therefore, it will only be preferred when there is a strong clinical indication, especially where there is a need to define pus collection in the neck or when the patient is not responding to surgical management (27,28).

Wong et al. recommended the use of ultrasound machines with high-frequency probes over CT scans for these patients as it also has the capacity to delineate moderate to large pus collections in the neck without the risk of radiation exposure (15).

## Conclusion

The management of cervicofacial cellulitis in pregnant patients may be challenging; however, the perception of the changes that occur in these subjects, implications in therapeutic decisions, and robust multidisciplinary care can ensure good outcomes.
